# Genomic Prediction of Testcross Performance in Canola (*Brassica napus*)

**DOI:** 10.1371/journal.pone.0147769

**Published:** 2016-01-29

**Authors:** Habib U. Jan, Amine Abbadi, Sophie Lücke, Richard A. Nichols, Rod J. Snowdon

**Affiliations:** 1 Department of Plant Breeding, IFZ Research Centre for Biosystems, Land Use and Nutrition, Justus Liebig University, Heinrich-Buff-Ring 26-32, 35392 Giessen, Germany; 2 NPZ Innovation GmbH, Hohenlieth, 24363 Holtsee, Germany; 3 Norddeutsche Pflanzenzucht Hans-Georg Lembke KG, Hohenlieth, 24363 Holtsee, Germany; 4 School of Biological and Chemical Sciences, Queen Mary University of London, Mile End Road, London E1 4NS, United Kingdom; Julius Kuehn-Institute (JKI), GERMANY

## Abstract

Genomic selection (GS) is a modern breeding approach where genome-wide single-nucleotide polymorphism (SNP) marker profiles are simultaneously used to estimate performance of untested genotypes. In this study, the potential of genomic selection methods to predict testcross performance for hybrid canola breeding was applied for various agronomic traits based on genome-wide marker profiles. A total of 475 genetically diverse spring-type canola pollinator lines were genotyped at 24,403 single-copy, genome-wide SNP loci. In parallel, the 950 F1 testcross combinations between the pollinators and two representative testers were evaluated for a number of important agronomic traits including seedling emergence, days to flowering, lodging, oil yield and seed yield along with essential seed quality characters including seed oil content and seed glucosinolate content. A ridge-regression best linear unbiased prediction (RR-BLUP) model was applied in combination with 500 cross-validations for each trait to predict testcross performance, both across the whole population as well as within individual subpopulations or clusters, based solely on SNP profiles. Subpopulations were determined using multidimensional scaling and *K*-means clustering. Genomic prediction accuracy across the whole population was highest for seed oil content (0.81) followed by oil yield (0.75) and lowest for seedling emergence (0.29). For seed yieId, seed glucosinolate, lodging resistance and days to onset of flowering (DTF), prediction accuracies were 0.45, 0.61, 0.39 and 0.56, respectively. Prediction accuracies could be increased for some traits by treating subpopulations separately; a strategy which only led to moderate improvements for some traits with low heritability, like seedling emergence. No useful or consistent increase in accuracy was obtained by inclusion of a population substructure covariate in the model. Testcross performance prediction using genome-wide SNP markers shows considerable potential for pre-selection of promising hybrid combinations prior to resource-intensive field testing over multiple locations and years.

## Introduction

Genomic selection (GS) is a modern breeding approach whereby genome-wide single-nucleotide polymorphisms (SNP) marker profiles are used to estimate individual breeding values of untested genotypes [1–3]. This novel biometrical approach was initially proposed in animal breeding [4] but is actively gaining currency for the improvement of various complex traits in plant breeding [5–8]. In GS, genome-wide markers are used that capture a distribution of genetic effects, from small to large, and thus, potentially accounts for a majority of the genetic variance for a given trait [9]. Thus, GS approach can be used without prior information on the effect of individual markers. Instead a ‘black box’ approach is adopted where combined marker effects are estimated [10].

GS in plant breeding is considered more challenging than in animal breeding due to the complex nature of genotype-by-environment interactions and their strong influence on plant reproduction [11]. On the other hand, genomic prediction can potentially shorten the breeding cycle by enabling early selection and increased selection intensity. In combination with potentially higher selection accuracy for traits with low heritability, this can ultimately boost genetic gain in comparison to conventional selection [12–13]. The implementation of cost-effective screening systems for high-density, genome-wide SNP markers makes genomic prediction approaches increasingly attractive [14–15].

The ridge regression best linear unbiased prediction (RR-BLUP) approach [4, 16] is becoming a method of choice in genome-wide prediction models due its computational ease combined with a high accuracy in predicting both polygenic and even non-polygenic traits. In crop plants RR-BLUP has been applied in various practical and experimental crop breeding scenarios [7, 17–20]. The development of GS models generally involves the use of a training population (TP) and a validation or prediction population (VP) [1, 21]. Both genotype data (molecular markers) and phenotype (field) data are collected from the individuals of the training population, while the validation population is used to test the performance (accuracy) of a statistical prediction model based solely on genotype data. Selection accuracy in GS can be affected by various factors including trait genetic architecture, linkage disequilibrium (LD) between the markers and quantitative trait loci (QTL), the number of markers, the size of the training population and the genetic relatedness of the training and validation populations [22–24]. A GS model for which the selection accuracy is equivalent or better in comparison to conventional selection, typically based on field performance in multi-environment evaluations, can potentially improve the selection gain by improving selection intensity or reducing the generation interval. In cases where a trait can only be accurately assessed using expensive phenotyping strategies, GS can also reduce costs for a given selection gain in a breeding programme.

*Brassica napus* L., commonly known as canola, oilseed rape or rapeseed, is one of the world’s most important oilseed crops because it delivers a rich source of high-quality edible oil and animal feed as extracted seed meal [25–27]. In Europe, the oil from winter-sown oilseed rape is also widely used as a sustainable biofuel [28]. The allopolyploid *B*. *napus* formed only a few thousand years ago [29] from spontaneous inter-specific hybridisations between turnip rape (*Brassica rapa*; AA, 2n = 20) and cabbage (*Brassica oleracea* CC, 2n = 18), and the gene pool of modern breeding materials is narrow due to this restricted genetic background and strong selection for essential seed quality traits [30–32]. According to Cowling [33], the effective population size of spring-type canola grown in Australia is just N_e_ ≤11, reflecting a huge loss of genetic diversity compared to the progenitor species *B*. *rapa* and *B*. *oleracea* [27].

Hybrid breeding has been instrumental in the exploitation of heterosis for yield gain and yield stability in plant breeding [34]. Due to its well-defined pollination control systems, *B*. *napus* can be used successfully for hybrid seed development [35–36]. On the other hand, the relatively narrow genetic diversity in modern breeding pools restricts the heterotic potential [37]. In comparison to classical hybrid crops like maize, in which genetically distinct heterotic pools have been established over many decades of hybrid breeding, there are no such clear heterotic pools available within canola germplasm. Development of new heterotic pools within adapted germplasm types, particularly through marker-assisted introgressions of novel germplasm from the diploid progenitors or other exotic gene pools, is an important strategy to overcome this problem [38–42]. Nevertheless, even in the absence of heterotic pools, genomic prediction of testcross performance is a highly promising method in canola breeding to select promising germplasm for advancement into male-sterile maternal lines or fertility restorers [43]. Recently genomic prediction has been demonstrated for estimation of testcross performance in various crops, for example in maize [7–8, 44], sugar beet [45], and rye [46].

In a hybrid breeding programme, efficient selection of the most promising combinations between male and female parental lines is a vital step to avoid expensive field testing of poor performing hybrids [20]. This becomes particularly important in crops like canola where the absence of distinct genetic pools prohibits a *per se* assumption of heterotic potential between any two potential hybrid parents. Various studies have reported methods for optimum exploitation of heterosis in crop breeding using both morphological and molecular marker data [47–51]. Piepho [52] described how the performance of untested hybrids can also be predicted effectively using genomic selection methodology.

In hybrid breeding system, male inbred lines are crossed with genetically distant ‘testers’ and general combining ability (GCA) and specific combining ability (SCA) values are estimated. The variances of GCA and SCA depend on the kind of gene actions involved. GCA includes additive effects of the total variances which make up the major portion of variances whereas SCA refers to the non-additive gene actions mainly comprising dominance and epistatic deviations. Information on GCA plays an important role in a breeder’s decision making to identify a viable hybrid [53]. GCA information has been used recently in various genome-wide prediction studies [20, 47]. In situations where GCA variance is predominant compared to SCA variance, prediction of hybrid performance based on parental GCA effects is an accurate approach [54]. Experimental studies in hybrid canola revealed that variance due to GCA is more pronounced and mainly additive effects contribute to hybrid performance compared to SCA effects [55].

Technical difficulties associated with the development of male-sterile lines in canola generally lead breeders to choose relatively small panels of maternal lines. On the other hand, some of the most widely used male-sterility systems have the benefit that all known *B*. *napus* accessions are restorers, so that testcross performance with available maternal lines is an important selection criterion for breeding of pollinators. The restorer lines have nuclear genes and are able to restore fertility in hybrid crosses. The goal of the present study was to investigate prediction of the best possible parental combination of pollinators crossed with the two testers lines in a testcross performance for a number of important traits in spring canola based on genome-wide SNP profiles. In particular, our objectives were (1) to examine strategies for genomic selection of suitable parental combination for the use in canola hybrid breeding, (2) to explore the effect of training population sample size on the prediction accuracy, and (3) to evaluate the potential for genomic prediction of GCA effects contributing to canola hybrid performance.

## Materials and Methods

### Genetic material

The experimental materials comprised a diverse population of spring-type *B*. *napus* with double-low seed quality (low erucic acid, low glucosinolate content) from a commercial canola breeding programme. The materials were carrying introgressions from the diploid progenitors of *B*. *napus*. Two representative male sterile female testers (tester 1 and tester 2) from a pool of testers carrying the *Male Sterility Lembke* (MSL) sterility system (NPZ Lembke, Hohenlieth, Germany) were crossed with a total of 475 pollinators to generate seed from 950 F1 hybrids.

### Phenotype data

The 950 testcrosses were evaluated at four different locations across Denmark, Germany, Poland and Estonia during the 2012 growing season. The locations for the field trials are commercial plant breeding testing sites. No specific permissions are required to grow conventional oilseed rape on these agricultural locations. Commercial crops like oilseed rape are not endangered or protected species. Un-replicated trials were performed in each of the four locations for various traits of commercial importance including seed yield (dt/ha), oil yield (dt/ha), seed oil content (% volume per seed dry weight), content of total seed glucosinolate (GSL; μmol/g seed), seedling emergence (visual observation ranging from a minimum value of 1 to maximum 9), lodging resistance (visual observation ranging from a minimum value of 1 to maximum 9) and days to onset of flowering (DTF; measured as number of days from sowing until 50% flowering plants per plot).

The restricted maximum likelihood (REML) method was used to estimate variance components. A best linear unbiased estimate (BLUE) was made for each trait (S1 File). All calculations were performed using the statistical software package SPSS Statistics for Windows Version 22.0 (IBM Corp., Armonk, NY, USA). Variance estimates were used to calculate broad sense heritability (*H*^*2*^) following the method given in [56].

$$H^{2}\left(\%\right)=\;\left[\frac{\sigma^{2}{}_{g}}{\left(\sigma^{2}{}_{g}+\sigma^{2}{}_{\varepsilon }/n\right)}\right]\times 100$$

where *σ*^*2*^_*g*_ is the genotypic variance, *σ*^*2*^_*ε*_ is the estimated residual variance, and *n* is the number of locations (Table 1). Estimates of residual variance *σ*^*2*^_*ε*_ were divided by the number of locations (in this case four).

**Table 1:** Summary statistics for seed yield (dt/ha), oil yield (dt/ha), seed oil content (%), seed glucosinolate content (GSL; μmol/g), seedling emergence (visual observation scale 1–9; good = 9), lodging resistance (visual observation scale 1–9; good = 9) and days to onset of flowering (DTF) in field trials with 950 spring canola F1 testcross phenotypes in 4 independent field locations throughout Europe. *σ*^*2*^_*g*_: genetic variance, *σ*^*2*^_*ε*_: estimated residual variance, *H*^2^: broad sense heritability.

Traits	Mean	Min.	Max.	*σ*^*2*^_*g*_	*σ*^*2*^_*ε*_	*H*^2^(%)
Seed yield (dt/ha)	31.17	23.94	38.38	1.56	7.95	44
Oil yield (dt/ha)	14.54	4.5	24.55	0.56	0.95	70
Oil content (%)	48.41	44.08	52.73	1.91	0.81	90
GSL (μmol/g)	9.22	6.91	11.57	1.35	3.12	63
Emergence (good = 9)	6.66	4.53	8.8	0.048	0.41	32
Lodging resistance (good = 9)	7.16	5.05	8.65	0.119	0.47	50
DTF	171.26	160.23	182.28	0.808	5.26	38

In our genomic prediction analysis, we used GCA values as phenotype matrix on all the pollinator lines. Pearson’s correlation coefficients (r) were calculated from the BLUE values between all the traits.

### Genotype data

Each of the 475 pollinator lines was genotyped using the *Brassica* 60k SNP Infinium consortium array (Illumina Inc., San Diego, CA; USA). Genomic DNA was extracted from young leaf samples collected 20 days after sowing, shock frozen in liquid nitrogen and stored at -20°C until further processing. The DNA extractions were performed using a BioSprint 96 magnetic bead nucleic acid extraction robot (Qiagen, Hilden, Germany) according to the manufacturer’s instructions. After fluorometric quantification of DNA concentrations using a Qubit 2.0 fluorometer (Life Technologies, Darmstadt, Germany), samples were diluted to 20ng/μl in sterile double distilled water, and quality checks of all DNA samples were carried out by gel electrophoresis on a 96 capillary Fragment Analyser (Advanced Analytical, Ames, IA, USA).

Genotyping on the 60k *Brassica* SNP array was outsourced to TraitGenetics GmbH (Gatersleben, Germany). All called SNPs were mapped to the *Brassica napus* cv. Darmor-bzh reference genome [29] using the basic local alignment search tool (BLAST) with no mismatches permitted in the flanking oligonucleotides. All SNPs showing multiple BLAST hits or a non-random distribution were removed and a total of 28,286 single-position SNPs remained. Furthermore, markers with allelic frequencies smaller than 0.05 and markers having more than 20% missing values were removed (S2 File). A total of 24,403 unique, single-copy SNPs remaining after filtration were used in the subsequent genomic prediction of testcross performance.

### Determination of population structure

Genetic relatedness between different genotypes can be estimated using molecular markers [57]. In the absence of clearly defined heterotic pools in *B*. *napus*, we analysed genetic relatedness and population substructure among the parental lines using the genome-wide SNP data. Roger’s genetic distances [58] were calculated among pairs of inbred lines. A multidimensional scaling (MDS) analysis based on principal coordinate analysis (PCoA) was performed using the filtered panel of 24,403 unique, single-copy genome-wide SNPs. The software package *SelectionTools* [59; www.uni-giessen.de/population-genetics/downloads] was used in *R* [60] for PCoA and *K*-means clustering.

Clusters of genetically related individuals were identified using the *K*-means method, following the algorithm of [61]. A diagnosis of the optimal number of clusters in the dataset was performed using the method described by [62].

### Scenarios for the genomic prediction of breeding values

**Fig 1:**
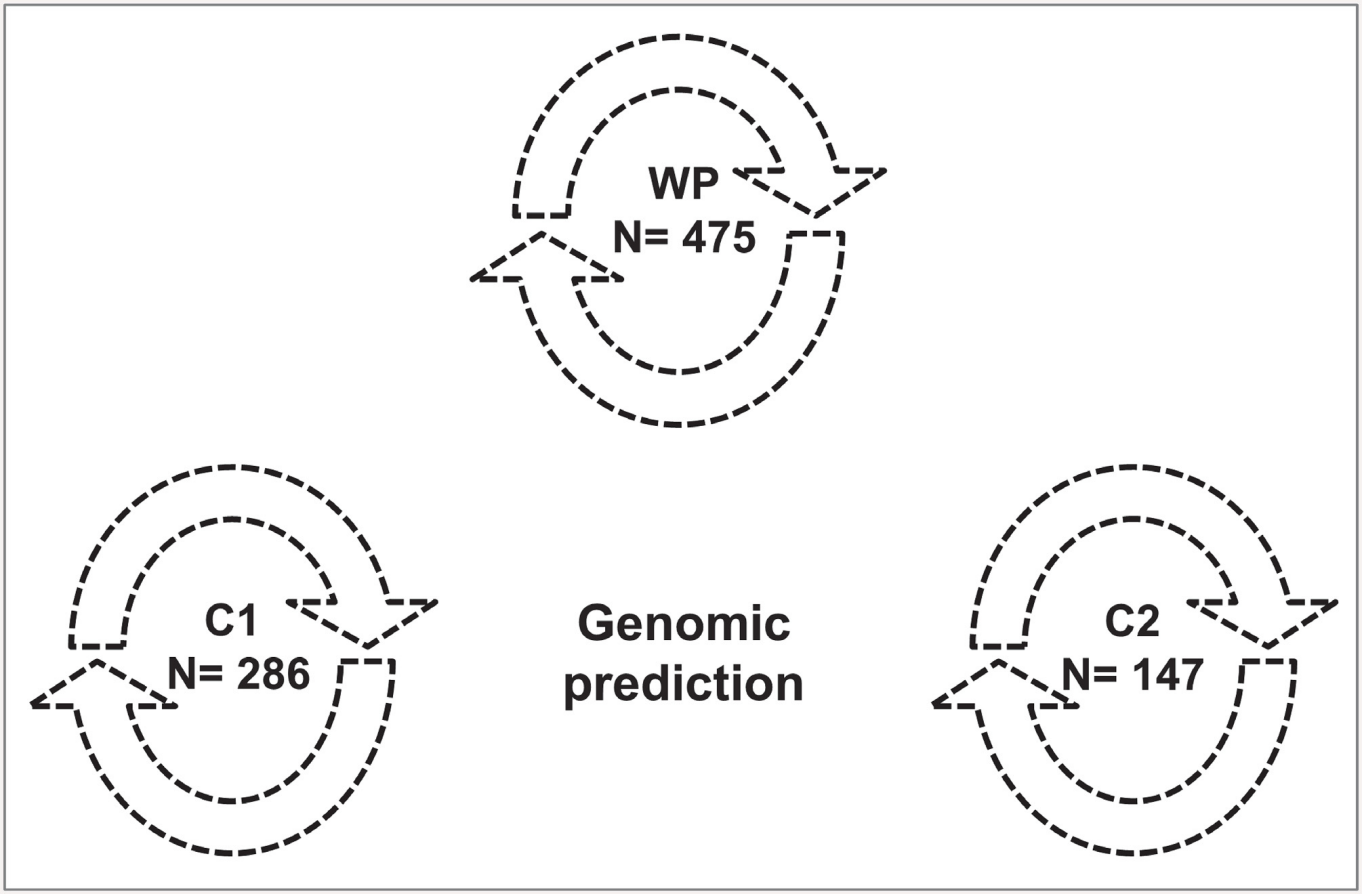
Genomic prediction across the whole population (WP) and genomic predictions within cluster 1 (C1) and cluster 2 (C2) separately are represented by dotted circular arrows. Fig 1 illustrates three independent genomic scenarios.

Three independent scenarios based on the population structure were applied to estimate marker effects by genomic prediction. In scenario 1, the genomic prediction was performed across the whole population (WP). We further tested the prediction accuracy across the whole population using a model that included the population substructure as a covariate. To investigate genomic prediction accuracy separately in the different genetic backgrounds of cluster 1 (C1) and cluster 2 (C2), respectively, we developed scenario 2 (prediction within C1) and scenario 3 (prediction within C2) (Fig 1). However, we did not directly compare prediction accuracies among these three prescribed scenarios due to confounding caused by their different sizes, and rather reported them separately. Results from predictions within subpopulation C3 alone are not reported due to the very small size of the test and validation populations in this case. In addition, we tested two more scenarios where we sampled the training sets across three subpopulations and validated these within the two main subpopulations C1 and C2, respectively.

### Genomic prediction using the RR-BLUP mixed model

Genomic prediction accuracies were estimated using the RR-BLUP model described by [4], [16], assuming the same distribution of marker effects across the whole-genome. The following model was used:

$$y=\mu +\overset{N_{m}}{\underset{i=1}{\sum }}X_{i}a_{j}+e,$$

where:

*y* is a *N* × 1 vector of phenotype (vector of BLUEs across locations);

*μ* is the overall mean;

*N*_*m*_ is the number of SNPs;

*a*_*j*_ is the effect of the *j*^*th*^ marker;

*X*_*i*_ is a *N* × 1 vector of genotypes (coded as 0,-1,+1) of the lines for each marker *j*, and variance of *a*_*j*_ is assumed to be uniformly distributed and is *σ*^2^_*G*_* / N*_*m*_ [4].

### Marker imputation and genomic prediction accuracy

Monomorphic SNPs and markers having more than 20% missing data were removed from the dataset. The rr-BLUP package in *R* [17] was used to estimate genomic predictions with the remaining missing data replaced using the default method (mean imputation). Genomic prediction accuracy, denoted as r_GPA_ was calculated for each trait. In some previous studies, prediction accuracies have been standardised by dividing the square root of the heritability to remove the corrected influence of heritability [54]. In our study, on the other hand, we report prediction accuracy (r_GPA_) as the Pearson correlation, r(y, ŷ), between the predicted values (ŷ) and observed BLUE values (y), using the rr-BLUP package [17]. Because the heritability is not considered for calculation of the BLUE values, it is not necessary or appropriate to correct for inferred heritability in the prediction model we applied.

### Model cross validation

For determination of the optimum composition of training population size, we tested the prediction accuracies for each of the seven traits in the whole population under incremental increase of the training population from 10% up to 90% of the 475 lines. Based on the results of this test (see below), the training population for all further analyses and scenarios testing was set up at 70% of the total lines in the given dataset. Hence, in each run, the dataset was divided into a random 70 percent training population (TP) containing both genotyped and phenotyped data, and 30 percent validation (VP) or prediction population having only SNP data and SNP effects with no consideration of phenotype values. For each scenario the data for each trait was cross-validated for 500 rounds and a mean value was subsequently considered.

## Results

### Population structure among pollinators

**Fig 2:**
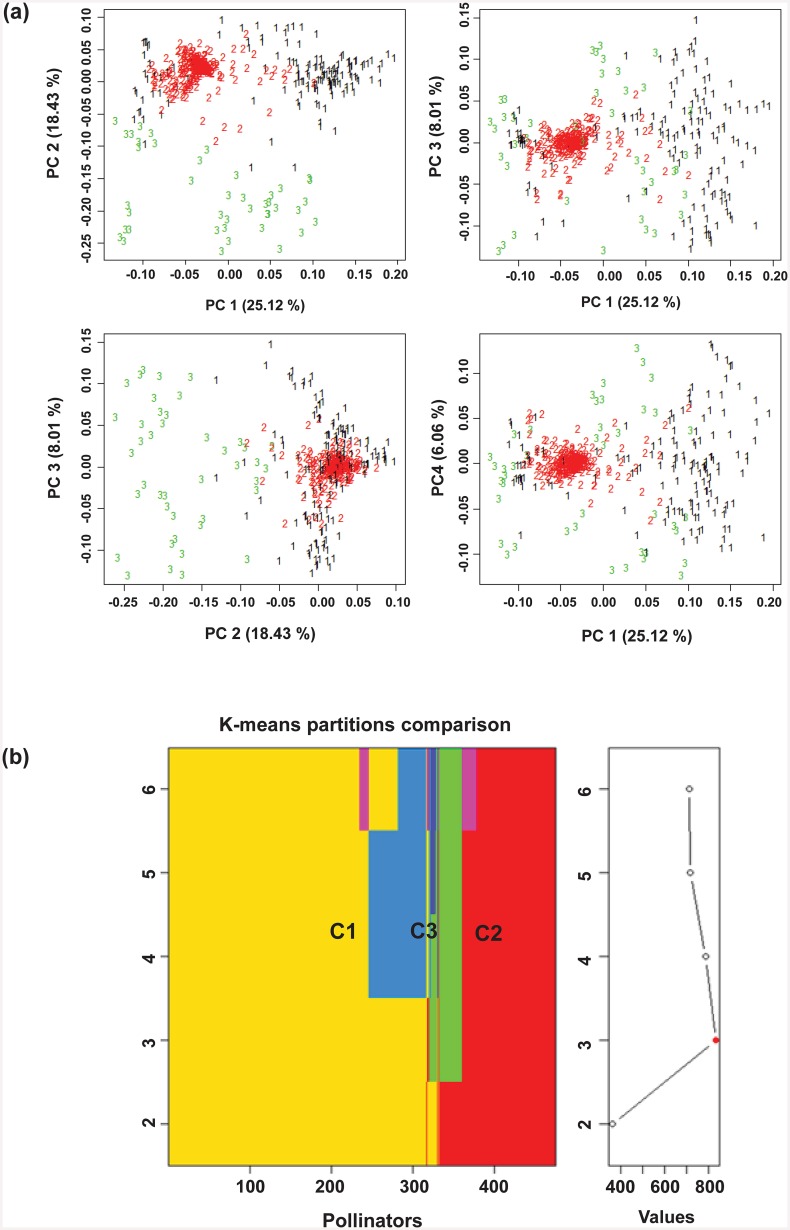
(a) Principal coordinates analysis (PCoA) among the population of 475 spring-type canola pollinators used for the testcross production. The PCoA is estimated using a panel of 24,403 filtered single nucleotide polymorphism (SNP) markers. Proportions of explained variance of principal coordinates 1, 2, 3 and 4 are given in parentheses. (b) K-means clustering of the 475 pollinator lines using the method of Caliński-Harabasz (1974) showing three clusters, i.e. cluster 1 (C1), cluster 2 (C2) and cluster 3 (C3) respectively. Fig 2 (a, b) shows sub-population structure in the dataset.

The results of the principal coordinate analysis (PCoA) between the parental inbred lines are shown in Fig 2a. The genetic variance explained by the first four principal coordinates comprised 25.12%, 18.43%, 8.01% and 6.06% respectively, making a total of 57.62%.

The PCoA indicated the existence of subpopulations within the dataset. The *K*-means clustering revealed a tendency to two main clusters and one relatively smaller cluster. This assumption was supported by the results of the Caliński-Harabasz [62] clustering, which also suggested three optimum clusters, as shown in Fig 2b. These are subsequently referred to as cluster 1 (C1; n = 286), cluster 2 (C2; n = 147) and cluster 3 (C3; n = 42), respectively.

### Broad sense heritability and variance components

**Fig 3:**
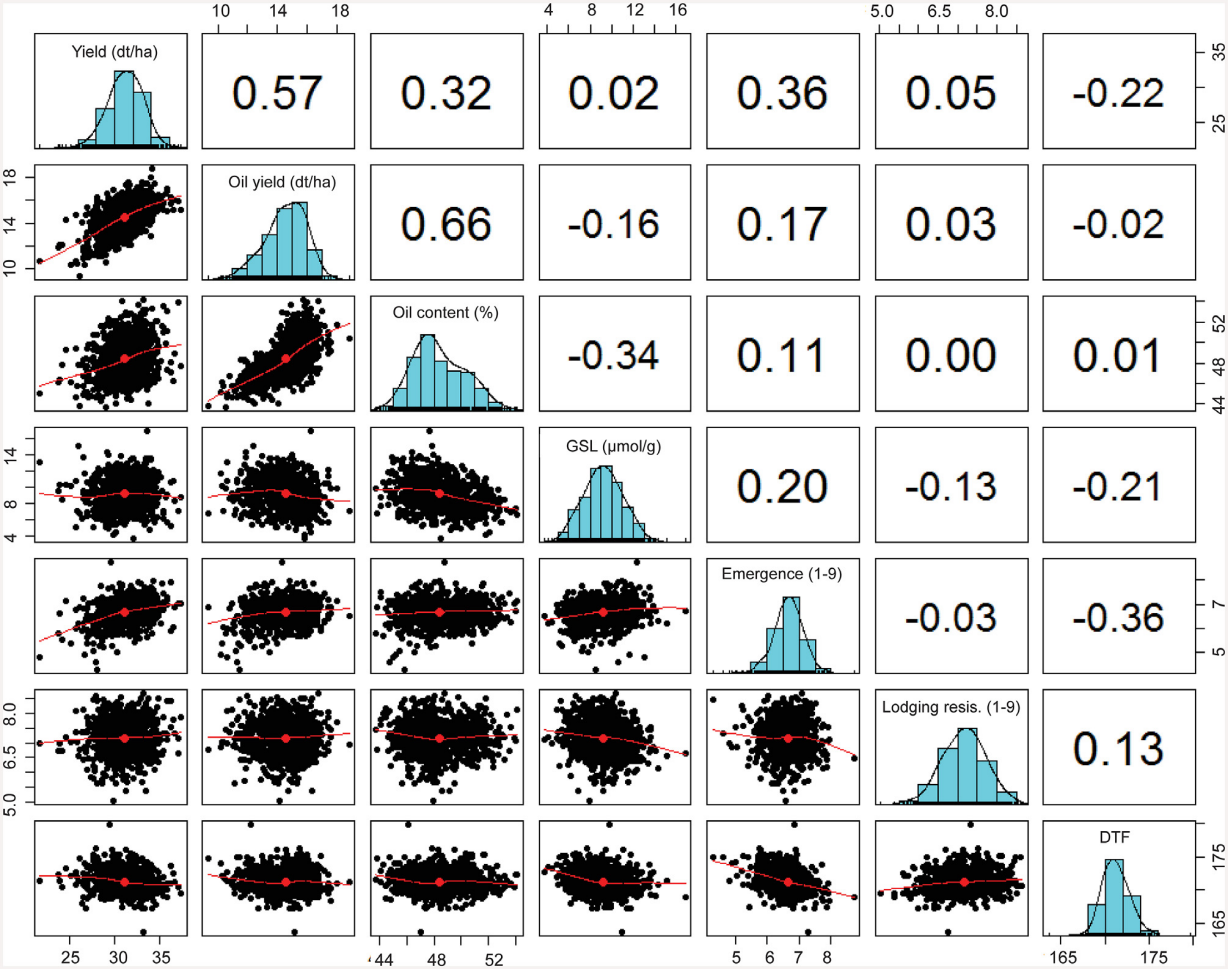
The Pearson’s correlations between all the seven traits. Highest positive correlation recorded between oil yield and seed oil content and lowest negative correlation recorded between seed oil content and seed GSL.

Broad sense heritabilities, along with summary statistics for all the traits under consideration, are shown in Table 1. Heritability values ranged from 32% for seedling emergence to 90% for seed oil content. Best linear unbiased estimates (BLUE) of each trait followed approximately the normal distribution expected for quantitative traits (S1 Fig). This was further confirmed by Q-Q plots drawn individually for each trait using R. The highest genetic variance was observed for seed oil content, while seedling emergence had the lowest genetic variance. As expected, positive correlations were observed between oil yield and seed oil content (r = 0.66) and between seed yield and oil yield (r = 0.57). Similarly, the expected highly negative correlation was observed between seed oil content and seed GSL (r = -0.34) Fig 3.

### Estimation of genomic prediction accuracy for different traits

**Fig 4:**
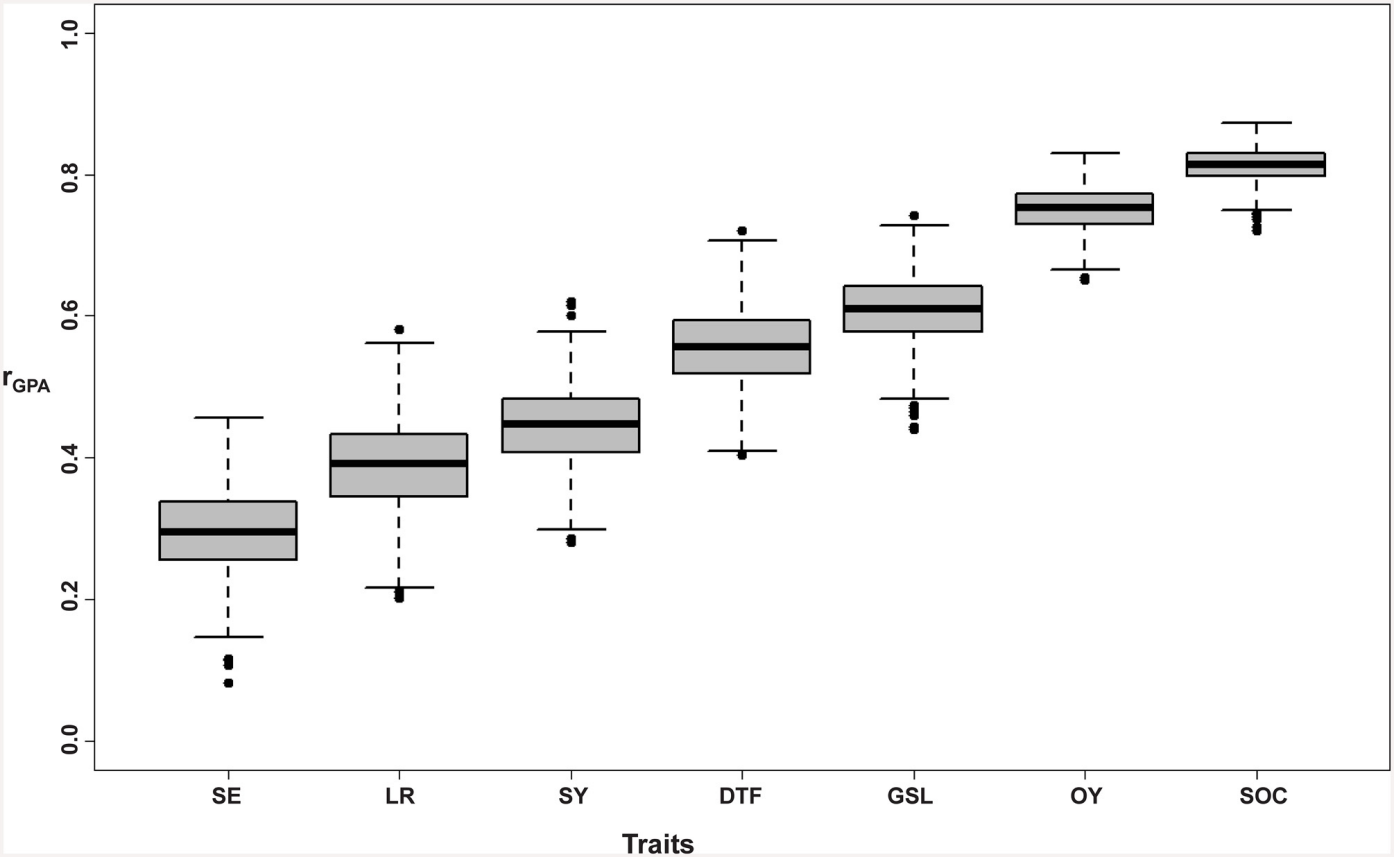
r_GPA_ across the whole test population for seedling emergence; SE, lodging resistance; LR, seed yield; SY, days to flowering; DTF, seed glucosinolate content; GSL, oil yield; OY and seed oil content; SOC, respectively. Fig 4 shows genomic prediction accuracies.

**Fig 5:**
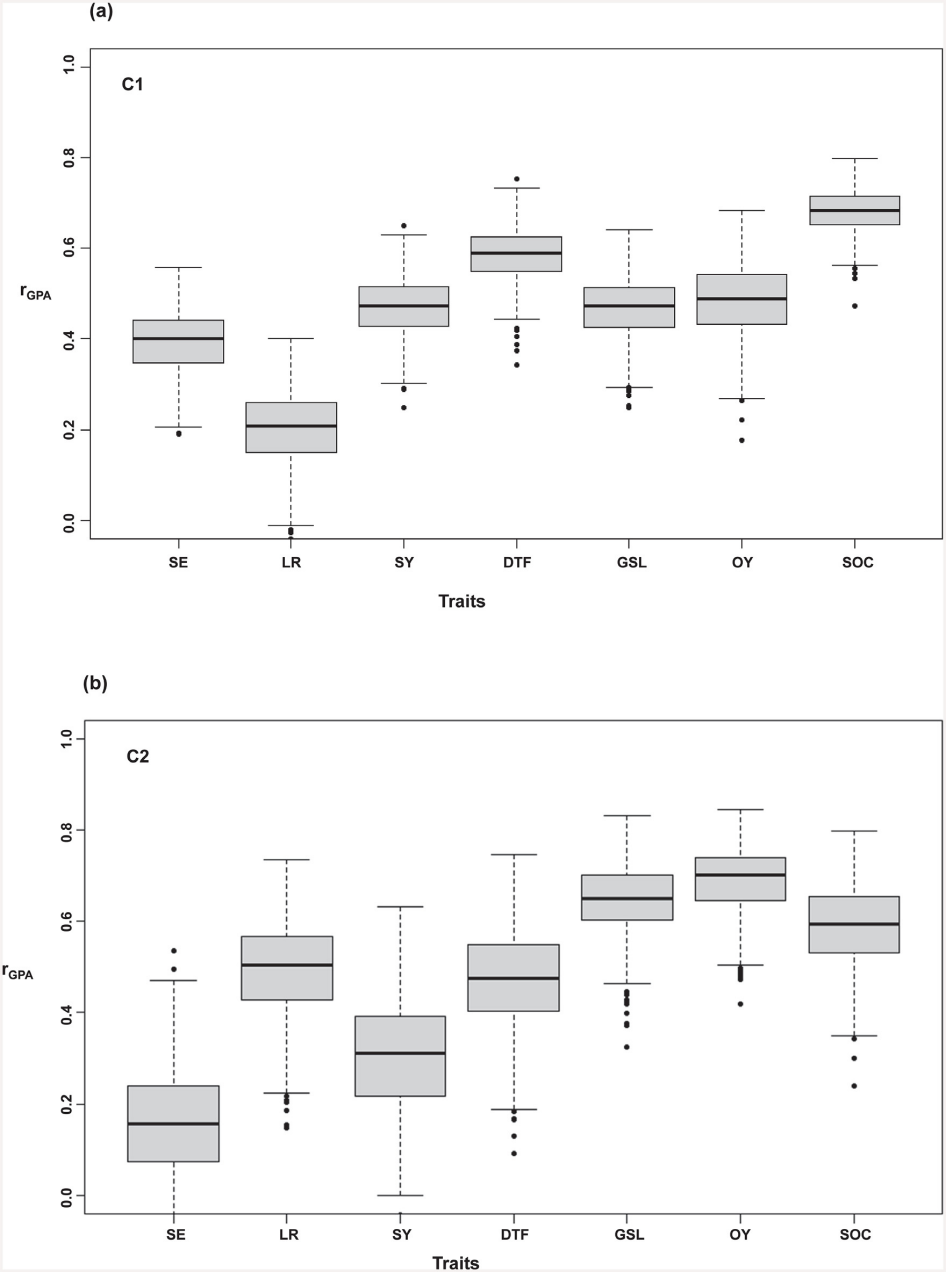
(a) Genomic prediction accuracies (r_GPA_) within cluster 1 (C1) for seedling emergence; SE, lodging resistance; LR, seed yield; SY, days to flowering; DTF, seed glucosinolate content; GSL, oil yield; OY and seed oil content; SOC, respectively. (b) Genomic prediction accuracies (r_GPA_) within cluster 2 (C2) for seedling emergence; SE, lodging resistance; LR, seed yield; SY, days to flowering; DTF, seed glucosinolate content; GSL, oil yield; OY and seed oil content; SOC, respectively. Fig 5 (a, b) shows genomic prediction accuracies across the two sup-populations.

1. Prediction across whole population: Fig 4 and Table 2 show the accuracies of genomic prediction for the respective traits based on GCA values, along with their respective standard errors for testcross performance using the whole population (WP) without consideration of population structure. For the seven traits considered, the highest prediction accuracy was recorded for seed oil content (r_GPA_ = 0.81) followed by oil yield (r_GPA_ = 0.75), seed glucosinolate content (r_GPA_ = 0.61), days to flowering (r_GPA_ = 0.56), seed yield (r_GPA_ = 0.45), lodging resistance (r_GPA_ = 0.39) and the least heritable trait, seedling emergence (r_GPA_ = 0.29). Scatter plots showing the correlations between true observed trait values and genomic predicted values for all the traits are shown in S2 Fig.
2. Predictions within subpopulations: Fig 5, S3 File show the independent prediction accuracies within subpopulations C1 and C2, respectively. Interestingly, an improved prediction accuracy (r_GPA_ = 0.39) was observed for the low-heritability trait seedling emergence within subpopulation C1, the largest subpopulation but also the narrowest in terms of genetic diversity. Predictions accuracies also improved for two other traits with low to moderate heritability, seed yield (r_GPA_ = 0.47) and DTF (r_GPA_ = 0.59) (Fig 5, S3 File). Similarly, within the second-largest subpopulation, C2, the prediction accuracies improved to r_GPA_ = 0.65 for GSL and r_GPA_ = 0.49 for lodging resistance, respectively (S3 File). For seed oil content and oil yield we observed no improvement in prediction accuracy within subpopulations compared to the whole population. Prediction accuracies in our additional two scenarios, where training set was taken across the three subpopulations and validated within C1 and C2, were either negative or close to zero for the majority of the traits (S4 File).

**Table 2:** Average prediction accuracies (r_GPA_) and standard errors (SE) for seed yield (dt/ha), oil yield (dt/ha), seed oil content (%), seed glucosinolate content (GSL; μmol/g), seedling emergence (visual observation scale 1–9; good = 9), lodging resistance (visual observation scale 1–9; good = 9) and days to onset of flowering (DTF) derived from 500 rounds of cross-validation across the whole-population.

Traits	Seed yield (dt/ha)	Oil yield (dt/ha)	Seed oil content (%)	GSL (μmol/g)	Seedling emergence (good = 9)	Lodging resistance (good = 9)	DTF
r_GPA_±SE	0.45±0.002^a^	0.75±0.001	0.81±0.001	0.61±0.002	0.29±0.002	0.39±0.003	0.56±0.002

^a^Approximate standard errors (SE) attached.

### Prediction accuracy and training population (TP) size

**Fig 6:**
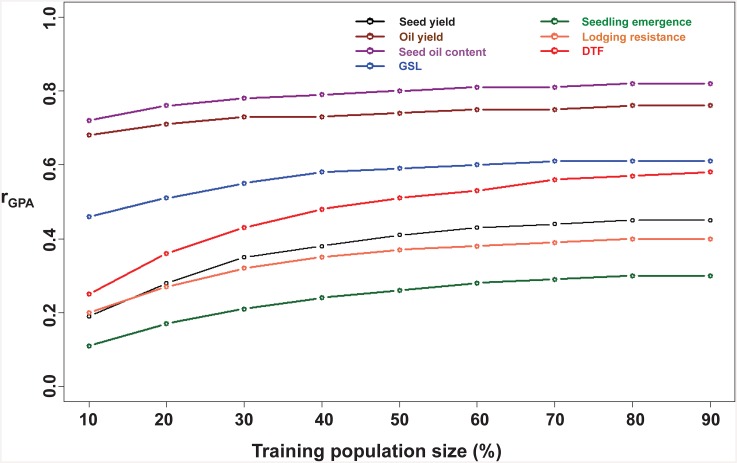
Influence of the size of the training population (TP; % of whole population size) on the genomic prediction accuracy (r_GPA_) for the seven traits seedling emergence, lodging resistance, seed yield, days to flowering (DTF), seed glucosinolate content (GSL), oil yield and seed oil content. Fig 6 shows the effect of training population size on genomic prediction accuracies.

As expected, increasing the size of the TP resulted in improvement of the genomic prediction accuracy (Fig 6). All the traits showed a plateau of prediction accuracy at a TP proportion of 80% except days to flowering, and only insignificant increases in accuracy as the TP size increased from 70% to 90%. We therefore, set an arbitrary TP size at 70% for all subsequent analyses and scenario testing.

## Discussion

The first investigation of the potential of genomic selection in *B*. *napus* breeding [63] investigated a relatively narrow set of winter oilseed rape breeding lines derived from 9 elite parental lines that were genotyped with only 253 SNP markers. To our knowledge, our study is the first report of testcross performance prediction in this important oil crop species. The population size, the represented genetic diversity and the number of SNP markers used for our analysis were all considerably larger than the previous study of [63].

We investigated genomic prediction accuracies for seven key agronomic traits including seed yield, oil content and quality related traits using a diverse population of spring-type canola. The RR-BLUP method used for the prediction modeling has been shown to be effective in accounting for both major and minor effect quantitative trait loci (QTL) in plant breeding [19–20, 63].

### Independent genomic prediction across the whole population

First we investigated genomic prediction accuracy for each trait across the whole-population based on GCA values. Taking the whole population under consideration, the lowest genomic prediction accuracy was estimated for seedling emergence and highest for seed oil content. The low genomic prediction accuracy for seedling emergence under scenario 1 may be explained by the low heritability and genetic variance for this trait. One strategy to increase prediction accuracy in such traits could be to combine these with other correlated high heritable traits in a multi-trait genomic prediction model which has been shown in a previous study [64]. In the case of seed oil content, the prediction accuracy remained high across the whole population. This is presumably due to the high heritability and the comparatively simple genetic architecture underlying this trait, where a few major QTL control maximum phenotypic variance [65, 66]. Oil yield showed the second highest prediction accuracy across the WP after seed oil content. This may be due to the strong positive correlation between these two traits. In our prediction analysis, genomic prediction accuracies based on additive genetic effects were higher in majority of the traits within the two tester pools.

Riedelsheimer et al. [47] and Saatchi et al. [67] reported that population substructure might affect genomic prediction accuracies. In our dataset, implementation of independent prediction within subpopulations increased prediction accuracies in specific subpopulations for low to moderate heritability traits like seed glucosinolate content, lodging resistance, DTF and seedling emergence. This is in line with the previous studies that reported higher prediction accuracies when genetically closely individuals were used in the TP and VP [22, 68]. The most straightforward explanation for such improvements might be that these traits are affected by variants at major-effect loci in some subpopulations that are rare or absent in the remainder of the materials. For some traits no improvement in accuracy were observed within subpopulations. This indicates that a large TP, in which the captured diversity strongly represents the diversity in the corresponding VP, may overcome the potential disadvantage caused by use of genetically distant individuals in the TP and VP. On the other hand, lowest prediction accuracies were obtained when a training set was derived from across all three subpopulations and validated within the two main subpopulations. This may indicate a lack of correspondence between the linkage phase of markers and QTL alleles across the different subpopulations. Adding a covariate to the prediction model which identified the clusters in the whole population did not improve the overall prediction accuracy for any trait. This scenario may be rather specific for canola, in which modern, adapted breeding pools have a particularly narrow genetic basis due to conscious selection for specific traits [31–33]. The situation is very different in maize or cattle, for example, where genetic differentiation among subpopulations or races are highly pronounced and population differences in gene and allele content are therefore often decisive [22, 68, 69]. We conclude that adjustment of prediction models on a case-by case basis in canola can potentially give small improvement in prediction of specific traits depending on the variance within a given breeding population.

For the high-value traits of seed oil content, oil yield and seed glucosinolate content, for which high heritabilities can be attributed to a good rank correlation among locations, we consistently obtained very high prediction accuracies in predictions across the entire population regardless of substructure. This may be further due to the modulating maternal influence of the two common male-sterile testers on embryo-related traits like seed size and oil content.

### Effect of TP sample size on genomic prediction accuracy

In simulation studies [68] as well as real datasets [8, 15, 67], it has been shown earlier that an increase in the training population size has a positive impact on the overall genomic prediction accuracy. In predictions across the entire test population, a TP comprising 70% of the overall population size (333 lines from 475) was sufficient to accurately predict the performance of the remaining lines for testcross performance. With the exception of flowering time, where the prediction accuracy still did not achieve a plateau even with 90% TP, only small or insignificant increases in accuracy were achieved with a TP proportion greater than 70%. The failure to achieve a plateau for flowering time suggests the presence of some accessions with distinctly different genetic control of flowering time. From a breeder’s viewpoint a smaller TP size is of course advantageous to reduce phenotyping costs. The most satisfying solution is the one in which adequate selection gains are achieved without surpassing current phenotyping costs.

### Genomic selection prospects in hybrid rapeseed

At the dawn of canola hybrid breeding various authors reported considerable heterosis in F1 hybrids [70–72]. The use of molecular markers to accelerate the differentiation of hybrid pools and investigate the genetic basis of heterosis [73–76] further increased hybrid performance; however levels of yield improvement seen in more classical hybrid crops like maize are still not achieved in canola. The development of heterotic pools in canola has made only slow progress in comparison to maize due to the generally low diversity within the species. The highly complex allopolyploid genome of *B*. *napus*, with multiple interacting homoeologous copies of almost all genes [29], increases the difficulty in prediction of individual gene actions [31]. Hence, genomic prediction of testcross performance could be a promising avenue for improving important traits without consideration of detailed *a priori* knowledge of their underlying genetics.

## Conclusions

The main purpose of genomic selection is the utilisation of large and inexpensive DNA marker datasets to bring an improvement to the mean performance of a certain population [77]. Seed yield, seed oil content and other polygenic traits are under the influence of complex genetic and biochemical interactions, and hundreds or thousands of small-effect QTL might be involved in their expression.

From a breeder’s perspective the implementation of genomic prediction is only worthwhile if equivalent or greater selection gain can be achieved with equal or reduced time and/or cost than using conventional selection methods (generally multiple-year, multiple-location field evaluations). Depending on the selection intensity, the results presented in this paper clearly demonstrates the value of performance predictions based on high-density SNP markers in hybrid canola. Relatively higher genomic prediction accuracies in the majority of the traits, based on the additive genetic effects in our study, indicate a lack of distinct heterotic pools in our sample. Even where no improvement on phenotypic selection gain is achieved through genomic prediction, the method is still of considerable value for traits like seedling emergence, where the very low heritability seedlots generated in multiple maternal environments combined with multi-location field evaluations. In such cases an increase in genetic gain might still be achieved if the early pre-selection approach enables a shortening of the breeding cycle. The results of our study suggest that prediction of testcross performance in canola breeding, based on genome-wide SNP markers, can be a powerful, fast and low-cost method to pre-select promising pollinators for combinations with available male-sterile maternal lines. Genomic testcross performance prediction can hence allow breeders to optimise the allocation of breeding resources. Despite the absence of clearly defined heterotic pools in canola, predictions within large subpopulations can in some cases improve prediction accuracy for selected traits with low heritability.

## Supporting Information

S1 Fig Trait distribution: a-g) Histograms and h-n) Q-Q plots of best linear unbiased estimates (BLUEs) for a,h) seed yield (dt/ha), b,i) oil yield (dt/ha), c,j) seed oil content (%), d,k) seed glucosinolate content (GSL; μmol/g), e,l) emergence (visual observation scale 1–9; good = 9), f,m) lodging resistance (visual observation scale 1–9; good = 9) and g,n) days to onset of flowering (DTF) in field trials from 8 independent locations. (TIF)

S2 Fig Scatter plots showing correlations between observed mean trait values (observed) and genomic predicted (predicted) for the seven traits evaluated. (TIF)

S1 File BLUE values for each trait. (XLSX)

S2 File *Spring type Brassica napus* (canola) genotype data (SNP) used in our analysis. (XLSX)

S3 File Genomic prediction accuracies (r_GPA_) for seven evaluated traits calculated independently within the two subpopulations C1 and C2. (XLSX)

S4 File Genomic prediction accuracies (r_GPA_) for seven evaluated traits calculated with a training set sampled from the three subpopulation and independently validated within the two subpopulations C1 and C2. (XLSX)
